# Complete Genome Sequence and Methylome of *Staphylococcus schleiferi*, an Important Cause of Skin and Ear Infections in Veterinary Medicine

**DOI:** 10.1128/genomeA.01011-15

**Published:** 2015-09-10

**Authors:** Ana M. Misic, Christine L. Cain, Daniel O. Morris, Shelley C. Rankin, Daniel P. Beiting

**Affiliations:** a Department of Pathobiology, School of Veterinary Medicine, University of Pennsylvania, Philadelphia, Pennsylvania, USA; b Department of Clinical Studies, School of Veterinary Medicine, University of Pennsylvania, Philadelphia, Pennsylvania, USA

## Abstract

*Staphylococcus schleiferi*, a Gram-positive and coagulase-variable organism, is an opportunistic human pathogen and a major cause of skin and soft tissue infections in dogs. Here, we report the first *S. schleiferi* genome sequence and methylome from four canine clinical isolates.

## GENOME ANNOUNCEMENT

*Staphylococcus* is an important genus of Gram-positive bacteria that are both commensal and pathogenic for humans and animals. The propensity of *Staphylococcus* species to develop antibiotic resistance, combined with the potential for zoonotic transmission (1–6), highlights the importance of understanding the genetic blueprint of *Staphylococcus* species that are commonly associated with infections in humans and animals. *Staphylococcus schleiferi* is a leading cause of drug-resistant pyoderma and otitis in dogs (7–10) and a source of rare but serious infections in humans (11–14).

Pulsed-field gel electrophoresis of *S. schleiferi* isolated from clinical samples at the Matthew J. Ryan Veterinary Hospital of the University of Pennsylvania showed that the population structure is dominated by four clonal clusters (15). Clonal cluster 1 accounts for the majority of clinical isolates, and 90% of the strains from this cluster have been shown to be methicillin resistant. Clonal cluster 2 is more heterogeneous and contains both methicillin-resistant (70%) and methicillin-susceptible isolates. Representatives from clonal clusters 1, 2, and 3 were selected for whole-genome sequencing from a banked collection of *S. schleiferi* isolates obtained from canine skin and ear canal infections between 2002 and 2013 from patients of the Matthew J. Ryan Veterinary Hospital of the University of Pennsylvania.

Isolates were grown in LB broth at 37°C overnight to an optical density at 600 nm (OD_600_) of approximately 1.2. Unamplified *S. schleiferi* genomic DNA was isolated using a Qiagen Genomic-tip kit (Valencia, CA, USA) and was used to construct SMRTbell sequencing libraries (Pacific Biosciences, Menlo Park, CA, USA). Sequencing was carried out using P4-C2 chemistry on a PacBio RSII sequencing platform with one 120-min. movie per cell. Sequencing each strain on a single PacBio single-molecule real-time (SMRT) cell yielded between 45,000 and 87,000 reads, with an *N*_50_ read length of approximately 6.5 to 10 kb. The SMRT Analysis software suite (version 2.0.1) (Pacific Biosciences) was used for *de novo* genome assembly with the HGAP.3 module (16). The sequences for each strain assembled to form a single contig representing the bacterial chromosome, with a coverage depth ranging from 59 to 140×, with >99.99% consensus accuracy. The genomic size range was 2,419,762 to 2,528,396 bp, with a G+C content of 35.8 to 36.0%. Isolate 5909-02 was additionally found to carry one 24-kb conjugative plasmid (p5909-02). Manual curation was used to resolve gaps and achieve a closed genome. Analysis of the *S. schleiferi* methylomes using the RS_Motif_and_Modification_Detection.1 module revealed that isolates 2142-05, 5909-02, and 2317-03 possessed two type I restriction-modification systems, while isolate 1360-13 possessed a type III restriction-modification system. The National Center for Biotechnology Information (NCBI) Prokaryotic Genome Annotation Pipeline (PGAP) was used to annotate the genomes and plasmid deposited into the NCBI Genomes database (17). A detailed analysis of the *S. schleiferi* genomes and comparisons to other *Staphylococcus* species will be included in a future publication.

### Nucleotide sequence accession numbers. 

Genome sequences for *S. schleiferi* strains 1360-13, 2142-05, 5909-02, a plasmid from 5909-02 (p5909-02), and strain 2317-03 have been deposited in GenBank under accession numbers CP009470, CP009762, CP009676, KP670395, and CP010309. PacBio sequence data for strains 1360-13, 2142-05, 5909-02, and 2317-03 are publicly available in the Sequence Read Archive (SRA) under accession numbers SRX701735, SRX847734, SRX738682, and SRX971499. Methylome data are available in the SRA under accession numbers SRP047175, SRP052584, SRP049178, and SRP056633. The genomes are also available in the Pathogen Resource Integration Center (PATRIC) at https://www.patricbrc.org.
